# Multi-Domain SDN Survivability for Agricultural Wireless Sensor Networks

**DOI:** 10.3390/s16111861

**Published:** 2016-11-06

**Authors:** Tao Huang, Siyu Yan, Fan Yang, Jiang Liu

**Affiliations:** 1 State Key Laboratory of Networking and Switching Technology, BUPT, Beijing 100876, China; yansiyu@bupt.edu.cn (S.Y.); yfan@bupt.edu.cn (F.Y.); liujiang@bupt.edu.cn (J.L.); 2 Science and Technology on Information Transmission and Dissemination in Communication Networks Laboratory, Shijiazhuang 050081, China

**Keywords:** wireless sensor network in agriculture, multi-domain SDN, network survivability

## Abstract

Wireless sensor networks (WSNs) have been widely applied in agriculture field; meanwhile, the advent of multi-domain software-defined networks (SDNs) have improved the wireless resource utilization rate and strengthened network management. In recent times, multi-domain SDNs have been applied to agricultural sensor networks, namely multi-domain software-defined wireless sensor networks (SDWSNs). However, when the SDNs controlling agriculture networks suddenly become unavailable, whether intra-domain or inter-domain, sensor network communication is abnormal because of the loss of control. Moreover, there are controller and switch info-updating problems even if the controller becomes available again. To resolve these problems, this paper proposes a new approach based on an Open vSwitch extension for multi-domain SDWSNs, which can enhance agriculture network survivability and stability. We achieved this by designing a connection-state mechanism, a communication mechanism on both L2 and L3, and an info-updating mechanism based on Open vSwitch. The experimental results show that, whether it is agricultural inter-domain or intra-domain during the controller failure period, the sensor switches can enter failure recovery mode as soon as possible so that the sensor network keeps a stable throughput, a short failure recovery time below 300 ms, and low packet loss. Further, the domain can smoothly control the domain network again once the controller becomes available. This approach based on an Open vSwitch extension can enhance the survivability and stability of multi-domain SDWSNs in precision agriculture.

## 1. Introduction

With the popularity of the Internet of Things (IoT), wireless sensor networks (WSNs) are widely researched and change our life considerably [1]. Especially in precision agriculture, WSNs have played an important role [2,3]. With the sensor functions of real-time monitoring and tracking [4,5], it can collect distributed data, analyze yielding, monitor agriculture environment conditions, decrease agricultural cost, and improve farm production [6,7]. However, it is easy for sensors nodes in agriculture to dynamically join and disjoin this network due to sensors powering off or unstable wireless signals affected by bad weather, which affects network stability. Moreover, as the mobile autonomous vehicles carrying multi-sensors are applied in precision agriculture [8,9], the connectivity of sensor nodes dynamically change frequently; therefore, it has become more important to enhance the stability and reliability of sensor network communication.

In recent years, software-defined networks (SDNs) has been a hot subject of research, especially multi-domain SDNs [10,11] that control large-scale networks. SDNs separate the control plane from the data plane; therefore, it can not only improve network efficiency of the forwarding in data plane but can also reach the centralized control [12]. With the SDN’s popularity, SDNs based on wireless sensors have attracted considerable attention recently [13]. Sensor network scalability is so large that using a multi-domain controller manages each domain network. When a multi-domain SDN is applied in an agricultural WSN, there are many advantages, such as the extension of agricultural network management scalability, an increase in wireless resource utilization rate, an improvement in the flexibility of network management, and the ability to customize applications [14,15]. 

However, there is a problem as to how the reliability of multi-domain software-defined WSNs (SDWSNs) can be enhanced. In bad agricultural weather environments, wireless sensor networks are affected so seriously that the sensor link becomes unstable. Agricultural vehicle sensor networks include numerous dynamic mobile sensor nodes carried by vehicles, which means that mobile sensor nodes will frequently join and disjoin the network. Therefore, once the wireless link between the controller and switches is disrupted, or any domain controller leaving its network becomes unavailable, both intra-domain and inter-domain network communications become abnormal due to the loss of control [16], which will severely impact monitoring agriculture. Moreover, there is still the info-updating problem of SDN controllers and sensor switches when the domain controller becomes available again.

To resolve the reliability problems of multi-domain SDWSNs in precision agriculture mentioned above, many attempts have already been carried out, such as cluster controllers [17], local agents [18], and OpenFlow’s double pipelines [19]. However, there are still challenging issues: First, how can controller failure and perform failure recovery in agricultural intra-domains and inter-domains be detected as soon as possible? Second, how can info-updating, after the SDN controller becomes available again, be ensured? Third, how does the mechanism limit the flexibility and benefit of SDNs in agricultural WSNs?

Based on the above analysis, this paper proposes a new approach based on the Open vSwitch extension for multi-domain SDWSNs in agriculture. This new approach can enhance intra-domain and inter-domain network communication performance and info-updating when the controller connection is lost. In addition, the scalability and flexibility of SDNs can be maintained.

We developed a connection-state mechanism, a communication reliability mechanism on network L2 and L3, and an info-updating mechanism based on Open vSwitch. Through experiments, we show that, when a domain controller suddenly disconnects, the failure recovery time is short enough to guarantee an uninterrupted communication service in the agricultural intra-domain and inter-domain network, and the domain controller is smoothly recovered.

Specifically, the main contributions of this paper are as follows:

- This new approach focuses on intra-domain as well as inter-domain networks. It uses a proposed communication reliability mechanism that detects controller unavailability as soon as possible and keeps intra-domain and inter-domain communication normal after the controller becomes unavailable, which improves the reliability of multi-domain SDWSNs.
- The controller can access the network again and maintain both the info-updating of topology information in the controller and the rules in sensor switches.

The rest of the paper is organized as follows. Section 2 presents the communication reliability mechanism, followed by Section 3 where the implementation of the modules is described. These concepts are evaluated in Section 4, and related work is analyzed in Section 5. Finally, the paper concludes in Section 6.

## 2. Communication Reliability Mechanism

This section provides the design details of the proposed communication reliability mechanism. In multi-domain SDWSNs, each agricultural network domain including numerous sensor switches is controlled by its own domain controller. These domain controllers work cooperatively or independently to ensure normal communication of the intra-domain and the inter-domain in the network data plane. 

### 2.1. Communication Reliability Mechanisms of the Intra-Domain and Inter-Domain

**Figure 1:**
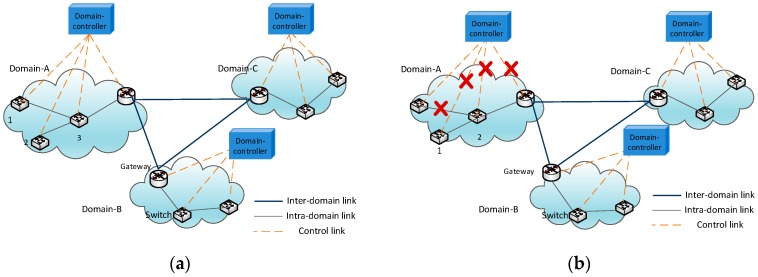
(**a**) Multi-domain software-defined wireless sensor networks (SDWSNs); (**b**) The link failure in a domain.

As shown in Figure 1a, each domain is linked together by each domain’s gateway, and the gateways and switches are controlled by the SDN domain controller in the agricultural sensor network. Once the wireless network becomes so weak that the sensor switches cannot connect to the domain controller, the domain controller will become unavailable, and the sensor switches lose control, which leads to communication interruption (Figure 1b). Therefore, this paper proposes communication reliability mechanisms to ensure normal communication when the SDN controller is unavailable, including a connection-state processing mechanism, an introducing self-learning mechanism, and an L3 mechanism into the data plane to keep the intra-domain’s and the inter-domain’s communication normal.

The connection-state detection mechanism: The connection state is the link state between the controller and each switch; therefore, this mechanism means, when a controller is suddenly unavailable, the data plane mainly depends on the connection-state processing mechanism to enter recovery mode quickly and smoothly. This connection-state detection mechanism can detect the real-time state by probes messages, such as the OpenFlow Echo message [20]. Failure recovery time depends on the detection time of controller failure; therefore, to get the latest connection state between the controller and sensor switch nodes as soon as possible, it dynamically adjusts the probe’s sending interval based on echo message’s Round-Trip-Time.

**Figure 2:**
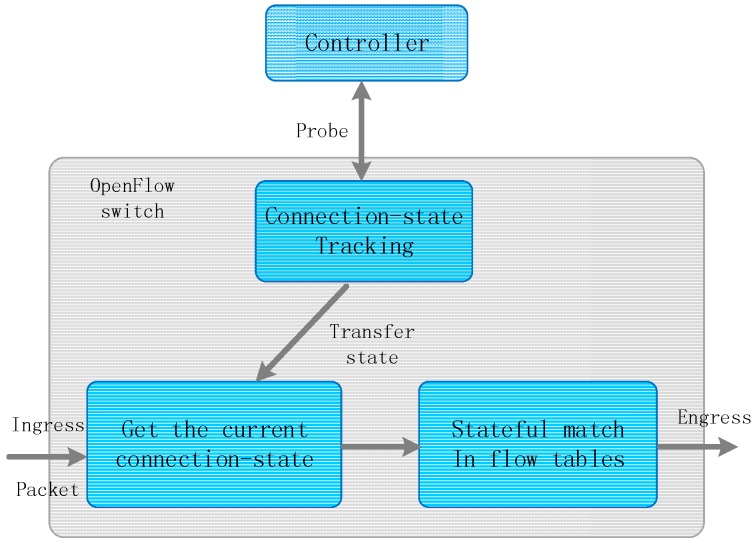
The connection-state processing mechanism. This mechanism uses a dynamic probe to track the connection-state and then transfers the real-time state to the incoming packet. The packet depends on the current state to execute an appropriate stateful match.

The stateful match mechanism: The connection states mainly consist of two states: connection-state and disconnection-state. Connection-state means that the domain controller informs the packet how to forward in switches and gateways. In contrast, disconnection-state means that the domain controller is unavailable and switches will enter failure recovery mode in sensor networks. To apply this connection state into both the controller and the switches, this paper extends a match field named connection-state in OpenFlow protocol. The OpenFlow entries can contain a connection state into their match fields. Therefore, as shown in Figure 2, after a sensor packet entering switches obtains the current connection state between the controller and this switch, it can match the stateful entries in the switch flow tables and then implement specific actions. For example, if a switch loses the controller control, the packets quickly match the entry, which includes a disconnection-state match field, and then enter disaster recovery mode.

The self-learning mechanism: When switches enter disaster recovery mode, it needs a self-learning mechanism to keep the agricultural intra-domain network normal. This self-learning mechanism is extended as a new OpenFlow protocol action, so the controller can use OpenFlow API to install an entry that consists of a flexibly self-learning action. The new self-learning mechanism is similar to the traditional switch L2-mechanism but can be invoked and inserted in the flow-table by a controller application, which maintains the flexibility of SDN. After a packet matches this entry consisting of a self-learning action successfully, then it will implement this self-learing action. This action process entails that the switch can parse the ingress port and mac address of the packet’s header. Then, a new entry will be generated and updated by this parsing information, which can forward a similar packet from the opposite direction; therefore, this new self-learning mechanism can ensure basic L2 communication in an intra-domain network when the controller becomes unavailable.

The L3 mechanism: When the network enters disaster recovery mode, inter-domain communication still needs to rely on domain gateways. However, after the domain gateways lose control of the controller, gateways do not know how to route the packets to another domain, seriously restricting the inter-domain communication. We designed the L3 mechanism in the data plane for two types of packets in case of controller failure. The first type of packet is that which will transfer from this domain to another domain network. This is designed to insert a stateful entry into gateways, which can forward this type of packet to a neighboring domain when the loss of controller happens. Then, the controller of the neighboring domain can correctly direct the packet. The second type of packet is that which will enter this domain network. For these packets, the gateway switch will implement an ARP mechanism to obtain the next hop mac address so that it can finish packet routing in case of controller failure. This paper proposed this simple but effective method to ensure normal inter-domain network communication when domain gateways lose control.

### 2.2. Info-Updating Mechanism

The domain sensor network loses control due to controller crashes or unstable wireless links in severe agricultural and weather conditions. However, when the SDN controller recovers from failure again or a new controller hands over the network, the controller will control the domain switches and gateways again in the agricultural sensor network. However, there is old staling information in the controller and switches. At this time, it becomes important to ensure the info-updating in both the domain controller and the switches because of the staling information when domain controllers control the network again.

When the unavailable controller recovers and manages the domain switches again, the controller will send switches some request messages to retrieve the latest information such as flow tables, entries, and topology. This information represents the latest network state, but the domain controller’s database still stores the past network information. Therefore, the controller database information, especially entries and topology, should be updated according to the latest network information regarding the issuing of request messages. 

**Figure 3:**
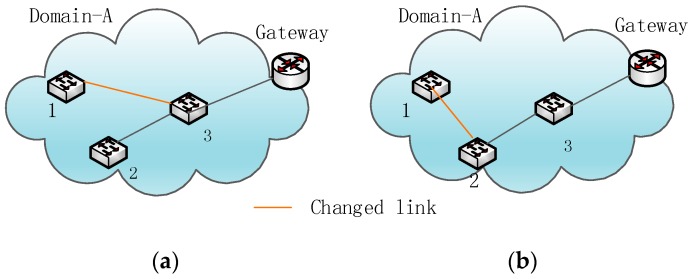
(**a**) The Domain-A topology before disaster; (**b**) the Domain-A topology after disaster.

Besides the above information updated in the controller database, the info-updating mechanism still needs to be used in updating and deleting stale entries in some sensor switch nodes because of the changing topology during controller failure. As shown in Figure 3, the connectivity relationship between switch-1 and other nodes changes, so some entries relevant to switch-1 should be updated. Therefore, when the controller controls the domain network again, it will use the LLDP protocol to retrieve the latest topology and compare this topology to the old topology before failure [21]. Then, it can determine the changing switch nodes and relevant entries to implement the detection of validity. For the stale entries detected, the domain controller will send OpenFlow messages to update and delete them. 

## 3. Module Implementation

To implement the proposed mechanism of Section 2 and easily build the prototype, this paper makes these mechanisms modular based on the OpenFlow Switch and OpenFlow protocols. Therefore, this section mainly describes how to implement the communication reliability module and the info-updating reliability module. 

### 3.1. Communication Reliability Modules

According to the description of the communication reliability mechanism, the communication reliability module should consist of three functions: connection-state processing, intra-domain reliability, and inter-domain reliability. To implement them, the communication reliability modules should include three modules, which are described below.

#### 3.1.1. The Module of Connection-State Processing

**Figure 4:**
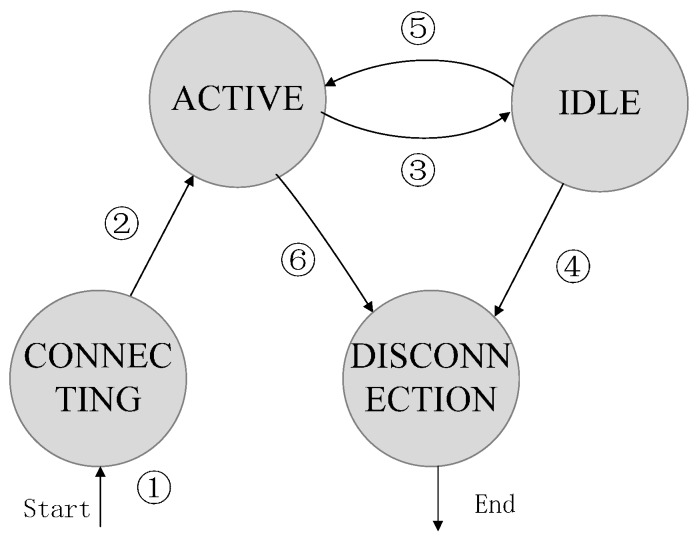
State transition. The switch can enter an appropriate state when the connection state between the controller and the switch changes.

This module mainly extends a stateful match field of the OpenFlow protocol and the OpenFlow Switch so that the sensor packets can match different stateful entries according to the states of the controller. The connection-state module is implemented by a finite state machine, which can clearly realize the state-jump function. As shown in Figure 4, the finite state machine consists of four states: CONNECTING, ACTIIVE, IDLE, and DISCONNECTED. In these states, both ACTIVE and IDLE belong to the connection state, which means that the domain controller is available. The states of CONNECTING and DISCONNECTED belong to the disconnection state, which means that the domain switches or the gateway still has not connected the controller successfully, which also means that the switches lose control of the domain controller.

As shown in Figure 4, at the beginning, the state machine enters the ACTIVE state from the CONNECTING state when the OpenFlow connection channel is built between the domain controller and the switch (see step 1 and step 2 in Figure 4). Then, the switch will send an echo message as a probe to track the real-time connection state between the domain controller and the switch. To obtain the current state as soon as possible, we dynamically adjust the echo message transmission interval and the timeout period according to the latest Round-Trip-Time of the echo message. Then, the state machine may enter IDLE from ACTIVE, or DISCONNECTION from IDLE, because of the echo message timeout (see steps 3 or 4 in Figure 4). Of course, if the SDN domain controller actively leaves the domain sensor network in agriculture, the state machine can directly enter DISCONNECTION from ACTIVE (see step 6 in Figure 4). Then, based on above mechanism, when coming from the switch port, the packet will obtain the current connection state via the finite state machine. The packet with the connection state will match the specific entry that contains the stateful match filed, such as the connection state field and the disconnection state field (see Table 1), and the packet can then implement different actions and receive appropriate processing. For example, it can design a disaster recovery flow table by a self-learning mechanism when the packet matches the disconnection entry under controller-unavailable conditions. Therefore, the sensor packet can smoothly and quickly enter and be processed, whether the domain controller is suddenly unavailable or the domain controller manages the agricultural sensor network again. 

#### 3.1.2. The Module of Intra-Domain Communication Reliability

The intra-domain module mainly depends on a self-learning mechanism. We implemented the self-learning by extending the OpenFlow and OpenFlow Switch API. To ensure intra-domain reliability, the domain controller can proactively insert a disconnection entry with the self-learning action into each switch with, for example, “match: state = disconnection-state, action: self-learning” (see also the first entry in Table 1). The packet will match this entry successfully after the domain controller becomes unavailable, which ensures the basic self-learning and forwarding functions in the intra-domain agricultural network. Meanwhile, self-learning combines the advantages of both an OpenFlow pipeline and a traditional L2-switch. We can design the module to implement this self-learning function. First, if a packet executes a self-learning action, the OpenFlow switch will parse the packet header to retrieve the source mac address and InPort field; second, it will generate a new entry whose “dst-mac” match field is the same as the source mac and “Output” action is the same as the InPort value. Third, it will detect whether there is an entry similar to this new entry. If it exists, this old entry will be updated by this new entry. On the contrary, it will insert this new entry directly into a specific flow table. 

#### 3.1.3. The Module of Inter-Domain Communication Reliability

**Figure 5:**
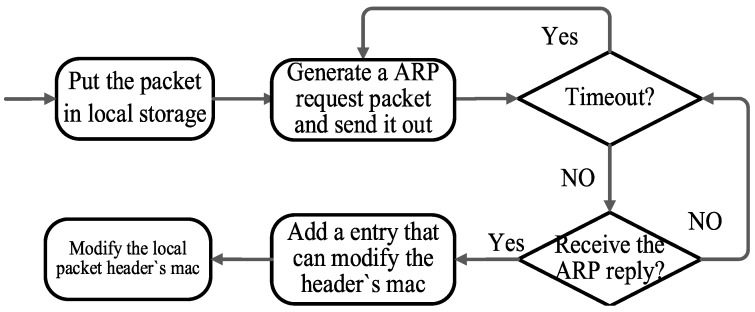
L3 module for the inter-domain network. Before the packet is routed in gateway and can’t get the destination mac, L3 module will send the ARP request to get the destination mac and generate a new entry to avoid triggering the ARP again.

This reliability module consists of two parts. For the packet out of the domain, it relies on a stateful entry in the gateway. When the SDN controller is available in an agricultural sensor network, the inter-domain module in the controller will insert a stateful entry in the gateway, which can forward the packet to a neighboring domain gateway for further processing. When the controller becomes unavailable, the entry will be valid and start to work. When the packet missing match in the gateway matches this entry and is forwarded to the neighboring domain, the controller of the neighboring domain will correctly direct the packet. For the packets arriving to this domain and entering this domain network, the destination mac of the packet will be modified before the packet is forwarded to the destination node by existing entries. If not matching any entry to modify the header of the packet, this gateway can implement an ARP module in the gateway so that the gateway can actively trigger an ARP request to retrieve the destination mac. The processing of the ARP module is shown in Figure 5. The ARP module ensures that the packet can be correctly modified and forwarded to the destination node. Inter-domain communication reliability can be realized by the above mechanisms and modules.

### 3.2. Info-Updating Reliability Module

After the sensor network and the domain SDN controller recover from failure, or new controllers hand over as primary controllers to control the network again, the controller should reconnect the switches and gateways and manage the domain network again in agriculture. However, the domain network states may have changed during the disaster period, such as in the topology, which leads to stale information in switches and the controller. Therefore, this paper proposes the design of an info-updating reliability mechanism module that can take charge of the updating of the information in switches as well as the controller’s database once the controller controls the domain network again.

#### 3.2.1. The Info-Updating Module in the Controller

There are two main types of information to be updated in the controller database—topology information and flow table information. First, when the sensor network recovers from network disaster, the topology may have changed during the disaster period; therefore, the controller will use LLDP protocol to obtain topology information. By sending LLDP packets requests to all switches, the controller can collect the current topology information such as the switch’s connection map relationship. Moreover, the controller will send request messages to switches to obtain the configuration information of the switches, such as port state. Then, this new topology information will update the old and stale information in the controller database so that applications such as the shortest-path-application can be used correctly based on these updated information. Second, it will update the flow table information in the controller database, such as entries and counters. In failure recovery mode, the switches adapt to the new topology and generate new entries in flow tables; therefore, the module of info-updating will send an OpenFlow status message to obtain the flow table information and replace this information in the controller database.

#### 3.2.2. The Module of Info-Updating in the Switches

When the controller controls the domain sensor network again, the topology may have changed so much that part of the entries in the switches have become stale and should be updated or deleted. The info-updating module can also update and delete these stale entries in the switches to achieve data plane connectivity and an auto-following strategy. The module mainly compares the latest topology information with the former topology. Then, it will find all of the switch nodes whose connection to other nodes has changed. Because the packets sent to these changing nodes will match the stale entries, these changing nodes will be unreachable. The module will update and delete these stale entries in the relevant switches directly. An example of info-updating reliability that clearly describes the entire process is given below. 

#### 3.2.3. An Example of Info-Updating Reliability 

**Figure 6:**
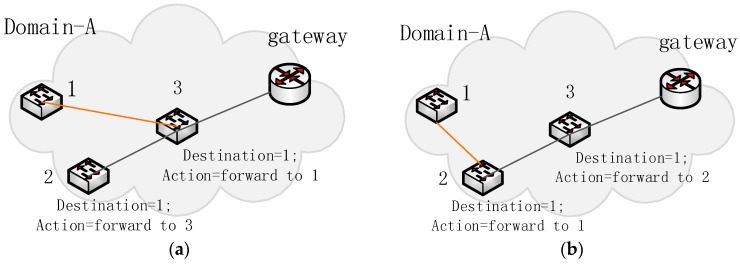
(**a**) Entries and topology before the controller failure; (**b**) Entries and topology after the controller failure. The orange link shows the connection relationship that changes. The entries that direct packets to switch-1 should be updated, too.

Let us suppose that the controller crashes due to bad agricultural weather and cannot control the sensor network. The controller then recovers from failure and manages the network again. Figure 6a is a part of the domain topology before the failure. Figure 6b is that part of the network topology after domain controller failure. The topology has significantly changed. This module can compare the two topologies and obtain changing switch nodes, such as switch-1. Switch-1 was connected to switch-3 before the domain controller failure happened, but now switch-1 connects with switch-2 directly; therefore, these entries related to switch-1 may be wrong. The entry whose destination is switch-1 in Figure 6a does not fit the new topology in Figure 6b, because the entry’s actions are wrong; therefore, the module will actively send the request message to switch-2 and switch-3 to update these entries according to the new topology. Therefore, when the domain controller manages the domain network again in agriculture, the info-updating reliability module can update the topology information in the controller database and reliably update the stale entries.

## 4. Experiment

In this section, we describe our experiment evaluation. We extended the RYU controller [22] and Open vSwitch (OVS) [23,24] as an OpenFlow switch in Mininet. To evaluate the reliability of the proposed mechanism of multi-domain SDWSNs in agriculture, we performed some experiments and analyzed the results.

If the links between the domain controller and switches are suddenly interrupted, the proposed mechanism should take more time to detect the failure because of continuous detection; therefore, this controller failure type will result in more complexity and damage than other failure types. To be more compelling, we selected this type of link failure as our experimental agricultural scenario.

There is a fail-secure mode in OVS that depends on a “NORMAL Port” action to simulate a traditional switch when the controller is not available [23]. To compare with our proposed mechanism, we use a fail-secure mechanism of OVS as a contrast test. Moreover, we mainly performed four experiments for failure recovery time, packet loss, throughput, and performance in different network scales. For clarity, we refer to the intra-domain experiment as AL2 and to the inter-domain experiment as AL3. For OVS’s own mechanism, which is a fail-secure mode, we refer to the intra-domain experiment as BL2 and to the inter-domain experiment as BL3.

### 4.1. Failure Recovery Time

**Figure 7:**
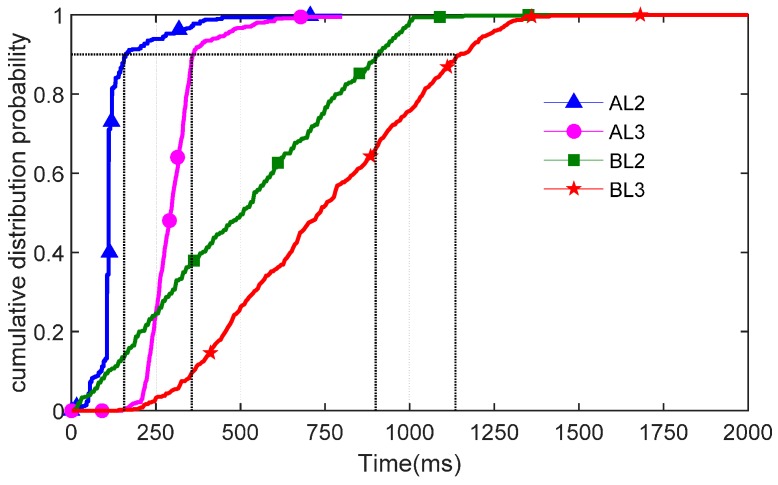
Failure recovery time of CDF (Cumulative Distribution Function).

The first experiment was to evaluate failure recovery time. We triggered the link failure between the domain controller and switches several times with different throughput. As shown in Figure 7, we measured the failure recovery time to obtain a failure recovery cumulative probability distribution.

The failure recovery cumulative probability distribution can show how much time the network requires from failure to normal communication with different mechanisms. The distribution was in excess of 1000 ms in OVS’s own mechanism in both intra-domain and inter-domain tests. However, AL2 and AL3, compared with than BL2 and BL3, yielded a shorter recovery time. The 90th percentiles of the intra-domain communication in AL2 were only about 100 ms, maintaining a low recovery time when controller failure occurred. For inter-domain sensor network communication, we used communication reliability mechanisms, such as ARP and stateful entries in the gateway. Because it required more time to obtain mac and change the packet header, the AL3 yielded about 300 ms. Therefore, the short intra-domain and inter-domain failure recovery time indicates that our proposed mechanism can make network communication normal as soon as possible when the domain controller becomes unavailable in agricultural sensor networks.

### 4.2. The Number of Consecutive Packet Losses

**Figure 8:**
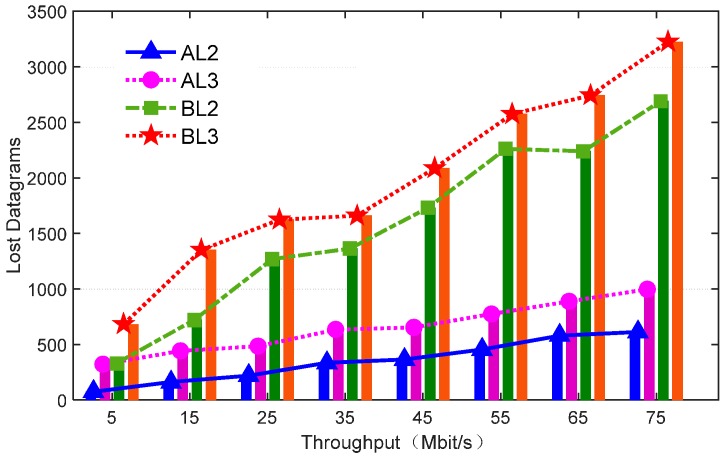
The number of consecutive packet losses during recovery time.

Figure 8 shows the packet losses during the recovery time among different mechanisms. The number of packets per Mbit UDP session was about 80.

In comparison with our proposed mechanism, BL2 and BL3 led to relatively severe packet losses. The high packet loss severely impacts sensor communication quality. In contrast, whether it is intra-domain AL2 or inter-domain AL3 in our mechanism, the number of packet losses is below 1000, which means that the packet loss rate is below 13%. We designed the controller connection-state tracking module to achieve a real-time state in a short period, so it can decrease the effect of domain controller failure.

### 4.3. The Throughput Stability

**Figure 9:**
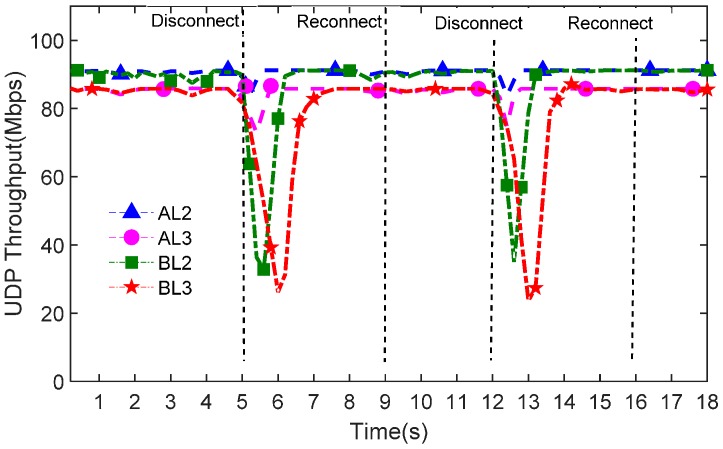
Instant throughput over time.

To show the throughput stability over time, we triggered the controller failure at the 5th and 12th seconds, as shown in Figure 9. Moreover, the domain controller manages the domain sensor network again after four seconds to show controller reconnection performance.

Figure 9 indicates that the throughput of mechanism BL2 and BL3 is less stable than AL2 and AL3. There are many fluctuations in the BL2 and BL3 throughput when domain controller failure occurs. On the contrary, our proposed mechanism maintains a stable throughput and a short failure recovery time in both intra-domain and inter-domain SDWSNs in agriculture.

More importantly, Figure 9 shows that the controller can smoothly reconnect the domain network and control all of the switches in the domain network again. After reconnection, the stale information is updated and the network can keep the stable sensor communication throughput; therefore, in our AL2 and AL3 mechanisms, it not only keeps the network communication stable when the controller becomes unavailable, but can also ensure that the controller manages the agricultural sensor network again smoothly.

### 4.4. The Relationship between the Topology Scale and Performance

**Figure 10:**
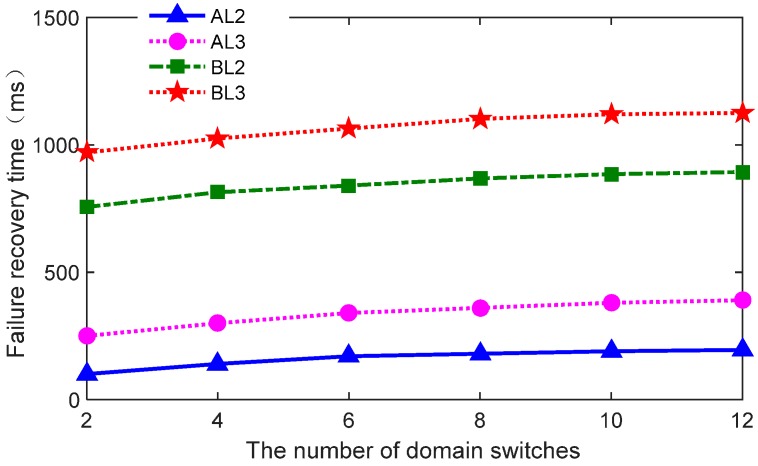
The relationship between the topology scale and performance.

Finally, we considered the relationship between topology scale and failure recovery performance when the controller becomes unavailable with sensor switches. Failure recovery time is measured as the number of area switches increases. The experiment result is shown in Figure 10.

Figure 10 indicates that there is slow and steady increase in failure recovery time even as the intra-domain and inter-domain switches increase several times; therefore, failure time is controlled when the sensor number increases. The reason is apparent because almost all of the sensor switches can simultaneously detect disconnection with the controller and enter failure recovery mode. More specifically, all of the sensor switches using the proposed connection processing mechanism send probe packets to detect the availability of the controllers by changing the connection state. Once a domain controller failure occurs and the domain sensor switches and gateway cannot connect to the failed controller, all of the switches and gateways can immediately detect the disconnection state at the same time so that these devices can enter failure recovery mode almost simultaneously. Therefore, in Figure 10, as the number of domain switches and gateways increase, failure recovery time changes very little. 

The small increase in failure recovery time is mainly affected by the number of switch nodes, the packet’s header parse, and the modification of switches. Whether in intra-domain switches or modified in the inter-domain gateway, the data packet will be parsed or modified to match the rules and forward, which inevitably leads to a small increase in failure recovery time. However, in general, the results show that our design does not cause a significant increase in failure recovery time, even though the topology scale increases.

## 5. Related Work

Recently, as the SDNs has stirred considerable attention, e.g., for control traffic balancing [25] and for the introduction of SDNs into WSNs. In [15] OpenFlow for wireless networks is extended and a sensor network is proposed. In [14], a stateful SDN for a wireless sensor network is defined. To extend network scalability, the study of a distributed SDN or a multi-domain SDN has become a hot topic. HyperFlow [18] and Onix [26] have made first attempts to use a distributed control plane to extend scalability and reliability. In [27], a west–east bridge mechanism for multi domain SDNs to cooperate with each other was proposed. Agricultural wireless sensor networks contain mass sensor nodes. Introducing multi-domain SDNs to WSNs can easily manage network resources and extend network scalability. 

However, it is easy for the data plane to lose control of the controllers when controller failure occurs. For example, the link between controller and switches link become unstable when the agriculture sensor network is faced with a bad natural environment. Moreover, the controller and switch info-updating also become a problem when the controller manages the network again. The attempts to resolve the problem are as follows: 

Controller cluster: The backup controller takes over the rule of the primary controller when the primary controller crashes or becomes unavailable. Optimizing the election mechanism [28] and the Optimizing Ravana Protocol [29] selects a slave controller to control the network when the main controller becomes unavailable. It is important to detect the controller status and controller role management quickly. Therefore, HyperFlow [18] records timestamps and checks it periodically. RMS [30] based on CPU utilization selects a controller as a health controller, but FCF-M [16] considers the controller load and distance to choose an appropriate alternative to decrease failover time. Moreover, compared with a complete controller crash, LegoSDN was proposed in [31] to protect controller-APPs against failure. However, the WSN link is unstable and severely affected by agricultural weather conditions; therefore, a controller cluster is not helpful when all of the controllers are disconnected from the data plane. In this paper, the network can work even when each controller suddenly becomes unavailable.

Data plane: In one study [20], a local agent installed in a data plane took over the controls of a network when the controller crashed. However, the experiment showed that throughput will decease by 40% after the controller becomes unavailable. Moreover, OpenFlow and Open vSwitch support a double pipeline, such as a normal port to perform traditional L2/L3 function under controller failure. This double pipeline mechanism has not only a long handoff time but also low flexibility and extendibility of SDNs. In contrast, this paper proposes a stateful match and an adaptive timeout to keep a short failure recovery time and flexibility of SDN. Moreover, single domain reliability is focused on in [32]—not inter-domain network reliability. More importantly, the authors of this study did not consider the info-update in the switches or the controller database, even though the controller managed the network again and there was stale information. This paper designed an info-updating mechanism for when the controller manages the network again. Furthermore, a new language named FatTire [33] ensures connectivity recovery for the link failure of the data plane, but it does not resolve the controller failure problem.

## 6. Conclusions

In multi-domain SDWSNs, each SDN domain controller manages its own domain sensor switches and sensor gateways in agriculture. Once the domain controller suddenly becomes unavailable, the data plane, including sensor switches and gateways, will lose control of the domain controller such that it leads to agricultural sensor network reliability problems in both inter-domain communication and intra-domain communication. For the above problems, this paper proposes connection-state tracking and a stateful match mechanism, an ARP mechanism, and an info-updating mechanism for agricultural multi-domain SDWSNs. Via these mechanisms, the experimental results show that, when the domain controller becomes unavailable, whether it is inter-domain or intra-domain, sensor switches can enter failure recovery mode as soon as possible so that there is stable throughput and a short failure recovery time below 300 ms. During the failure period, the packet loss is very low. Moreover, the domain can smoothly control the domain network when the controller becomes available again. Therefore, our proposed approach can enhance the survivability and stability of multi-domain SDWSNs in precision agriculture.

More importantly, besides the agricultural scenario, it is worth considering that the proposed reliability mechanism can be applied in other network scenarios to improve the reliability of SDNs. Therefore, more work and evaluation of the proposed design is needed.

## Figures and Tables

**Table 1:** Entries with different connect state match fields.

Flow Table	Match	Actions
0	connect_state = disconnection	self-learning, go to flow Table 1
	connect_state = connection	go to flow Table n
